# Infection With the Severe Acute Respiratory Syndrome Coronavirus 2 (SARS-CoV-2) Delta Variant Is Associated With Higher Recovery of Infectious Virus Compared to the Alpha Variant in Both Unvaccinated and Vaccinated Individuals

**DOI:** 10.1093/cid/ciab986

**Published:** 2021-12-18

**Authors:** Chun Huai Luo, C Paul Morris, Jaiprasath Sachithanandham, Adannaya Amadi, David C Gaston, Maggie Li, Nicholas J Swanson, Matthew Schwartz, Eili Y Klein, Andrew Pekosz, Heba H Mostafa

**Affiliations:** Johns Hopkins School of Medicine, Department of Pathology, Division of Medical Microbiology, Johns Hopkins School of Medicine, Baltimore, Maryland, USA; Johns Hopkins School of Medicine, Department of Pathology, Division of Medical Microbiology, Johns Hopkins School of Medicine, Baltimore, Maryland, USA; National Institute of Allergy and Infectious Disease, National Institutes of Health, Washington D.C., USA; W. Harry Feinstone Department of Molecular Microbiology and Immunology, The Johns Hopkins Bloomberg School of Public Health, Baltimore, Maryland, USA; Johns Hopkins School of Medicine, Department of Pathology, Division of Medical Microbiology, Johns Hopkins School of Medicine, Baltimore, Maryland, USA; Johns Hopkins School of Medicine, Department of Pathology, Division of Medical Microbiology, Johns Hopkins School of Medicine, Baltimore, Maryland, USA; W. Harry Feinstone Department of Molecular Microbiology and Immunology, The Johns Hopkins Bloomberg School of Public Health, Baltimore, Maryland, USA; W. Harry Feinstone Department of Molecular Microbiology and Immunology, The Johns Hopkins Bloomberg School of Public Health, Baltimore, Maryland, USA; Johns Hopkins School of Medicine, Department of Pathology, Division of Medical Microbiology, Johns Hopkins School of Medicine, Baltimore, Maryland, USA; Department of Emergency Medicine, Johns Hopkins School of Medicine, Baltimore, Maryland, USAand; Center for Disease Dynamics, Economics, and Policy, Washington D.C., USA; W. Harry Feinstone Department of Molecular Microbiology and Immunology, The Johns Hopkins Bloomberg School of Public Health, Baltimore, Maryland, USA; Department of Emergency Medicine, Johns Hopkins School of Medicine, Baltimore, Maryland, USAand; Johns Hopkins School of Medicine, Department of Pathology, Division of Medical Microbiology, Johns Hopkins School of Medicine, Baltimore, Maryland, USA

**Keywords:** SARS-CoV-2, Delta, Alpha, cell culture, IgG

## Abstract

**Background:**

The severe acute respiratory syndrome coronavirus 2 (SARS-CoV-2) variant of concern (VOC) B.1.617.2 (Delta) displaced B.1.1.7 (Alpha) and is associated with increases in coronavirus disease 2019 (COVID-19) cases, greater transmissibility, and higher viral RNA loads, but data are lacking regarding the infectious virus load and antiviral antibody levels in the nasal tract.

**Methods:**

Whole genome sequencing, cycle threshold (Ct) values, infectious virus, anti-SARS-CoV-2 immunoglobulin G (IgG) levels, and clinical chart reviews were combined to characterize SARS-CoV-2 lineages circulating in the National Capital Region between January and September 2021 and differentiate infections in vaccinated and unvaccinated individuals by the Delta, Alpha, and B.1.2 (the predominant lineage prior to Alpha) variants.

**Results:**

The Delta variant displaced the Alpha variant to constitute 99% of the circulating lineages in the National Capital Region by August 2021. In Delta infections, 28.5% were breakthrough cases in fully vaccinated individuals compared to 4% in the Alpha infected cohort. Breakthrough infections in both cohorts were associated with comorbidities, but only Delta infections were associated with a significant increase in the median days after vaccination. More than 74% of Delta samples had infectious virus compared to <30% from the Alpha cohort. The recovery of infectious virus with both variants was associated with low levels of local SARS-CoV-2 IgG.

**Conclusions:**

Infection with the Delta variant was associated with more frequent recovery of infectious virus in vaccinated and unvaccinated individuals compared to the Alpha variant but was not associated with an increase in disease severity in fully vaccinated individuals. Infectious virus was correlated with the presence of low amounts of antiviral IgG in the nasal specimens.

## Teaser

The Delta variant displaced the Alpha and associated with increased symptomatic breakthrough infections. The recovery of infectious virus was significantly higher with the Delta in both vaccinated and unvaccinated groups. Recovery of infectious virus correlated with lower respiratory IgG levels.

Severe acute respiratory syndrome coronavirus 2 (SARS-CoV-2) genomic evolution led to the emergence of variants that are more transmissible, cause severe disease, and escape natural or vaccine induced protective immunity. Lineage B.1.1.7 (Alpha) was classified as a variant of concern (VOC) by the US Centers for Disease Control and Prevention (CDC) due to evidence of higher transmissibility and concern for more severe disease [1]. The Alpha variant, which was first detected in Southeast England in September 2020 [2], predominated new SARS-CoV-2 infections in the United Kingdom by December 2020, spread globally, and rapidly became the major lineage in the United States by April 2021 [3, 4] with estimates that it was 50% more transmissible and had a 43–90% higher reproduction number compared to other SARS-CoV-2 lineages [5]. Although early reports found no correlation between Alpha and increased severity of disease [5], recent studies report an association with higher mortality [6] and risk of hospitalization [7].

The B.1.617.2 (Delta) variant displaced the Alpha in the United States after a nationwide decline in the total numbers of cases in June 2021 and became the most frequently sequenced lineage by July 2021 [8]. The Delta variant was classified as a VOC by the World Health Organization (WHO) in May 2021 due to notably increased transmissibility, even in the context of increasing percentages of fully vaccinated individuals in various communities. The Delta variant was associated with SARS-CoV-2 outbreaks and breakthrough infections in vaccinated individuals [9, 10], but vaccines continue to limit severe disease, hospitalization, and death [11–13].

The reasons for increased transmission of both the Alpha and Delta are unclear, but one proposed hypothesis is that these variants are able to attain higher viral loads in the respiratory tract of infected individuals [5, 14]. Some reports have found an association between the Alpha and Delta variants and higher viral loads in the upper respiratory tract [5, 6, 10]. Quantifying these differences is particularly important in vaccinated individuals, where the Delta variant has been associated with comparable cycle threshold (Ct) values in vaccinated versus unvaccinated patients [15]. Additionally, studies have assessed the recovery of infectious virus and neutralizing antibody levels [16, 17]. We used a large cohort of samples characterized by whole genome sequencing between January 2021 and September 2021 to compare the clinical characteristics, Ct values from upper respiratory specimens, recovery of infectious virus, and nasal SARS-CoV-2 immunoglobulin G (IgG) for Delta and Alpha variants.

## METHODS

### Ethical Considerations and Data Availability

Research was conducted under Johns Hopkins IRB protocol IRB00221396 with a waiver of consent. Remnant clinical specimens from patients that tested positive for SARS-CoV-2 after standard of care testing were used for this study. Whole genomes were made publicly available at GISAID.

### Specimens and Patient Data

The clinical specimens used for sequencing were nasopharyngeal (for symptomatic patients) or lateral mid-turbinate nasal swabs (for asymptomatic patients) after standard of care diagnostic or screening testing was performed during inpatient and outpatient encounters across the Johns Hopkins Medical System (which encompasses 5 acute care hospitals and more than 40 ambulatory care offices). In addition, specimens were obtained through standard of care screening and testing services performed by the health system at several long-term care facilities in the State of Maryland as well as through mobile outreach clinics in local neighborhoods. Molecular assays used for diagnosis include RealStar® SARS-CoV-2 reverse transcription polymerase chain reaction (RT-PCR) (Altona Diagnostics), Xpert Xpress SARS-CoV-2/Flu/RSV (Cepheid), NeuMoDx SARS-CoV-2 (Qiagen), Cobas SARS-CoV-2 (Roche), ePlex Respiratory Pathogen Panel 2 (Roche), Aptima SARS-CoV-2 (Hologic), and Accula SARS-CoV-2 assays (ThermoFisher Scientific) [18–21]. Molecular diagnosis of SARS-CoV-2 at Johns Hopkins Hospital laboratory began on 11 March 2020 [22], and whole genome sequencing for identifying circulating SARS-CoV-2 variants started as early as March 2020 as well [22]. Surveillance efforts for VOCs were increased at the end of October 2020 to monitor the evolution of SARS-CoV-2. Each sample in our cohort represents a unique patient. Table 1 shows the numbers of samples used for each part of the study.

**Table 1.** Clinical Charts and Samples Used for the Study

	B.1.2	Alpha	Delta
Total patients	377	1482	785
Samples with Ct values			
Total	224	564	251
Breakthrough			
Total		46	87
Asymptomatic		20	15
Known days to symptoms		26	72
Unvaccinated			
Total	200	470	134
Asymptomatic	9	28	18
Known days to symptoms	191	442	116
Samples with cell culture			
Total		154	128
Breakthrough		46	39
Unvaccinated	58	95	77
Samples with respiratory IgG			
Breakthrough		43	24
Unvaccinated		30	17

Abbreviations: Ct, cycle threshold; IgG, immunoglobulin G.

### Clinical Data Analysis

Patient data were bulk extracted from a data warehouse that contains all encounter-related information from hospital and outpatient visits to any Johns Hopkins Medical Institutions Facilities. Coronavirus disease 2019 (COVID-19) related hospitalization, intensive care unit (ICU) level care, and mortality were confirmed by manual chart reviews. Symptom-onset date was a questionnaire included in the chart and was answered by the patient at the specimen collection time. Symptomatic illness was defined based on the ordering clinician and was also a questionnaire included in the clinical charts. Vaccination status was determined through local vaccination first. If there was no local information entered (this includes both documented vaccinations given and self-reports documented in the system) registries from across the state that are pulled into the electronic medical records were then searched as were registries from nearby states (particularly Washington D.C.) and insurance registries. Notably, Johns Hopkins has an agreement with the State of Maryland and with CRISP (Chesapeake Regional Information System for our Patients) to provide vaccination status and positive infection information for all Johns Hopkins Patients. In general, the majority of the vaccinated patients’ population with known vaccine types in our cohort received the Pfizer/BioNTech (72.6%), followed by the Moderna mRNA-1273 (26.8%) and the J&J/Janssen COVID-19 vaccines (0.6%). Vaccine breakthrough infections were based on the CDC definition of positive test results more than 14 days post the second shot for Pfizer/BioNTech BNT162b2 and Moderna mRNA-1273 or 14 days after the J&J/Janssen shot.

### Ct Value Analysis

To ensure comparable Ct values for viral load analyses, samples with available Ct values after clinical testing was performed with the most commonly used diagnostic platform at Johns Hopkins (the NeuMoDx SARS-CoV-2 https://www.fda.gov/media/136565/download) were included in this study. Specifically, Ct values of the N gene were compared.

### Amplicon-based Sequencing

Specimens were extracted using the Chemagic™ 360 system (Perkin Elmer) following the manufacturer’s protocol. In total, 300 µL of sample was extracted and eluted in 60 µL elution buffer. Sequencing and data analysis were performed as previously described [4, 22]. Briefly, libraries were prepared in 96 well plates using the ARTIC protocol (V3). Nanopore reads were basecalled with MinKNOW and demultiplexed with guppybarcoder that required barcodes at both ends. Alignment and variant calling were performed with the artic-ncov2019 medaka protocol. Thresholds were set to a minimum of 90% coverage and 100 mean depth. Mutations were visually confirmed with Integrated Genomics viewer (IGV) (Version 2.8.10), clades were determined using Nextclade beta v 0.12.0 (clades.nextstrain.org), and lineages were determined with Pangolin COVID-19 lineage Assigner (COG-UK (cog-uk.io)).

### Cell Culture

Vero-TMPRSS2 cells were cultured and infected with aliquots of swab specimens as previously described for VeroE6 cells [23]. The presence of SARS-CoV-2 was confirmed by RT quantitative PCR (qPCR).

### ELISA

Undiluted respiratory samples were tested with the EUROIMMUN Anti-SARS-CoV-2 enzyme-linked immunosorbent assay (ELISA) (IgG) following the package insert (https://www.fda.gov/media/137609/download). The assay detects antibodies to the S1 domain of the spike protein of SARS-CoV-2 with a cutoff of <0.8 for negative results and ≥0.8 to <1.1 as borderline. The value 1.1 was used as a cutoff for respiratory specimen types such that values >1.1 were deemed positive.

### Statistical Analysis

Statistical analyses were conducted using GraphPad prism. The χ^2^ and Fisher exact tests were used for categorical variable comparisons, and *t*-test and Kruskal-Wallis 1-way analysis of variance (ANOVA) tests were used for comparing continuous independent variables.

## RESULTS

### SARS-CoV-2 Positivity and Variant Trends

**Figure 1.**
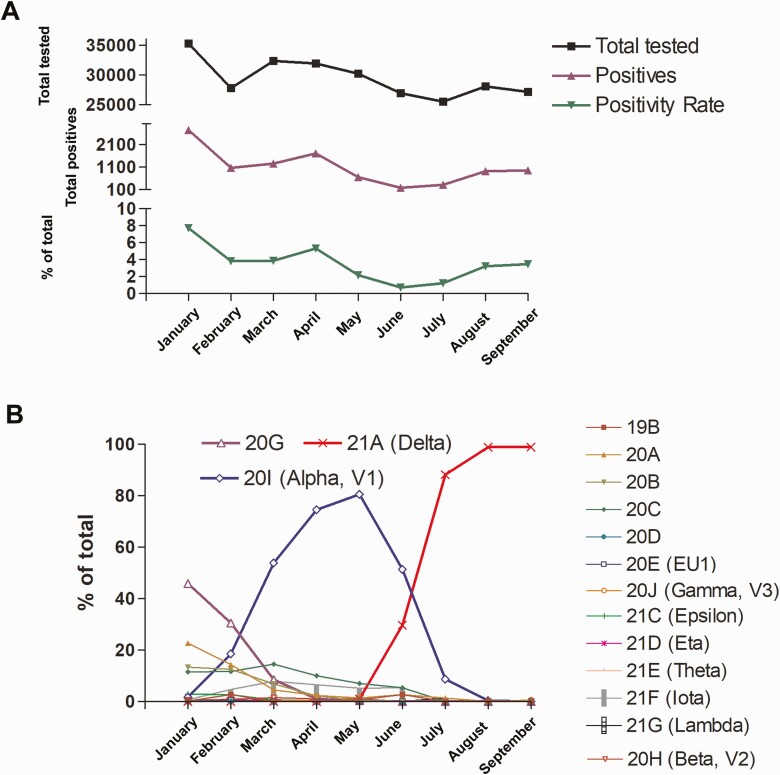
SARS-CoV-2 positivity and variant trends. Abbreviation: SARS-CoV-2, severe acute respiratory syndrome coronavirus 2.

Between January 2021 and September 2021, a total of 265 127 samples were tested at the Johns Hopkins Hospital Laboratory with positivity rates that declined from 7.7% in January to 0.7% in June with a notable increase in August and September to 3.2% and 3.5% (Figure 1A). Of 6151 genomes sequenced in this time frame, our data showed that the predominant circulating lineages (primarily B.1.2, clade 20G) were displaced by Alpha in late February [4], which were subsequently displaced by Delta at the end of June to constitute 99% of the circulating variants in August and September (Figure 1B). Other VOC and VOI were detected only infrequently during this time frame (Figure 1B).

### Patient Characteristics and Infection Outcomes in Alpha and Delta infections

Clinical chart reviews were performed for all Delta (785), Alpha (1482), and B.1.2 (377) infected patients diagnosed at Johns Hopkins laboratory from January 2021 to September 2021. The Delta variant was associated with a significant increase in confirmed breakthrough infections when compared to the Alpha variant (28.5% vs 4%, *P* < .00001, Table 2). There was a significant increase in the median days after vaccination for the Delta variant breakthroughs compared to Alpha variant breakthroughs (178.4 vs 20.1, *P* < .00001, Table 2). Delta variant infected patients were significantly different in race distribution when compared to the Alpha with a significant increase in infections in White race (*P* = .0003); however, Black patients predominated infections with both variants. An increase in COVID-19 related mortality and admissions was noted with the Delta variant (*P* = .01 and .003). However, the Delta infected group had a lower prevalence of certain comorbidities including lung, kidney, and heart disease (Table 2).

**Table 2.** Clinical and Metadata of the B.1.2, Alpha, and Delta infected patients

	B.1.2	Alpha	Delta	*P* Value: Alpha to B.1.2	*P* Value: Delta to Alpha
Total	377	1482	785		
Asymptomatic (%)	80 (21.2)	211 (14.2)	108 (13.76)	***.0015***	.7511
Symptomatic (%)	297 (78.8)	1257 (84.8)	675 (85.98)		
All positives after vaccination (%)	39 (10.3)	184 (12.4)	300 (38.2)		
Confirmed breakthrough (%)	2 (0.5)	59 (4)	224 (28.5)	***.0002***	***<.00001***
Median days after first dose (all positives after vaccination)	9.4	20.1	178.4	***<.00001***	***<.00001***
Median age (SD)	45 (21.8)	36 (21.3)	38 (22.4)	***<.00001***	.053
Female (%)	140 (37.1)	865 (58.4)	450 (57.3)	***<.00001***	.623
Male (%)	237 (62.9)	616 (41.6)	335 (42.7)		
Race					
Asian (%)	24 (6.4)	34 (2.3)	47 (6)		
Black (%)	108 (28.6)	860 (58)	492 (62.7)	***<.00001***	***.0003***
White (%)	209 (55.4)	416 (28.1)	333 (42.4)		
Other/unknown (%)	32 (8.5)	172 (11.6)	111 (14.1)		
Disease severity					
Mortality (%)	3 (0.8)	16 (1.1)	20(2.5)	.78	***.01***
Admission (%)	28 (7.4)	165 (11.1)	122 (15.5)	***.0372***	***.003***
ICU (%)	5 (1.3)	54 (3.6)	41 (5.2)	***.0204***	.08
Comorbidities					
Hypertension (%)	128 (33.9)	460 (31)	230 (29.3)	.2918	.41
Respiratory failure (%)	18 (4.8)	159 (10.7)	82 (10.4)	***.0002***	.88
Pregnancy (%)	24 (6.4)	118 (7.9)	53 (6.8)	.3295	.32
Lung disease (%)	83 (22)	358 (24.2)	124 (15.8)	.416	***<.00001***
Kidney disease (%)	40 (10.6)	204 (13.8)	82 (10.4)	.1238	***.024***
Immunosuppression (%)	68 (18)	251 (16.9)	117 (14.9)	.6462	.23
Diabetes (%)	50 (13.3)	235 (15.9)	108 (13.8)	.2301	.2
Heart failure (%)	17 (4.5)	120 (8.1)	44 (5.6)	***.0152***	***.03***
Atrial fibrillation (%)	15 (3.9)	65 (4.4)	43 (5.5)	.8869	.26
Smoker (%)	34 (9)	212 (14.3)	72 (9.2)	***.0063***	***.0004***
Cerebrovascular disease (%)	26 (6.9)	104 (7)	53 (6.8)	1	.86
Cancer (%)	125 (33.2)	318 (21.5)	175 (22.3)	***<.00001***	.67
Coronary artery disease (%)	55 (14.6)	236 (15.9)	93 (11.8)	.5786	***.009***

Statistics for ages and median days after vaccination were calculated by *t* test, and all other statistics were calculated by χ^2^ test.

Abbreviations: ICU, intensive care unit; SD, standard deviation.

The Alpha variant was associated with a significant increase in symptomatic infections when compared to the precedent B.1.2 lineage (84.8% vs 78.8%, *P* = .0015, Table 2). However, there was no similar increase from Alpha to Delta (*P* = .75, Table 2). A reduction in the median age, reduction in the male to female ratio, and an increase in infections in African-Americans were noted for the Alpha variant compared to the B.1.2 lineage (Table 2). When compared to the B.1.2, the Alpha variant showed a significant increase in COVID-19 related hospitalization and ICU level care but not mortality.

When vaccine breakthrough infection cases were compared to the unvaccinated patients in the Alpha and Delta groups, no significant differences in the likelihood of COVID-19 related hospital admissions were observed. However, the Alpha, and to a lesser extent the Delta, vaccine breakthrough groups were characterized by significantly higher immunosuppression and other comorbidities (Table 3). Comorbidities including hypertension, immunosuppression, cancer, and coronary artery disease were associated with vaccine breakthrough infections with the Alpha and Delta variants (Table 3). Vaccine breakthrough infections with the Delta variant were associated with a significantly higher percentage of symptomatic infections (84.4% vs 61%, *P* = .0002) and a marked increase in the median days after receiving the vaccine (195.7 days vs 77.3 days, *P* < .00001, Table 3).

**Table 3.** Clinical and Metadata of Delta and Alpha Vaccinated and Unvaccinated Patients

	Delta					Alpha					
	All Positives After Vaccination	True Breakthrough	Unvaccinated	*P* Value: All Positives After Vaccination to Unvaccinated	*P* Value: True Breakthrough to Unvaccinated	All Positives After Vaccination	True Breakthrough	Unvaccinated	*P* Value: All Positives After Vaccination to Unvaccinated	*P* Value: True Breakthrough to Unvaccinated	*P* Value/ Alpha to Delta True Breakthrough
Total	300	224	485			184	59	1298			
Median days after 1st dose (SD)		195.7 (47.6)					77.3 (27.9)				***<.00001***
Symptomatic (%)	252 (84)	189 (84.4)	423 (87.2)	.17	.28	148 (80.4)	36 (61)	1109 (85.4)	***.0424***	***<.00001***	***.0002***
Asymptomatic (%)	48 (16)	35 (15.6)	60 (12.4)			36 (19.6)	23 (38.9)	175 (13.5)			
Median age	47 (18.7)	46 (18.5)	30 (22.2)	***<.00001***	***<.00001***	53	51	34	***<.00001***	***.14***	.130
Females (%)	193 (64.3)	149 (66.5)	257 (53)	***.002***	***.0008***	112 (60.9)	42 (71.2)	753 (58)	.52	.06	.54
Males (%)	107 (35.6)	75 (33.5)	228 (47)			72 (39.1)	17 (28.8)	544 (41.9)			
Race											
Asian (%)	34 (11.3)	31 (13.9)	13 (2.7)			4 (2.2)	1 (1.7)	30 (2.3)			
Black (%)	83 (27.7)	53 (23.7)	211 (43.5)	***<.00001***	***<.00001***	73 (39.7)	13 (22)	787 (60.6)	***<.00001***	***<.00001***	.6
White (%)	161 (53.7)	124 (55.4)	172 (35.5)			86 (46.7)	38 (64.4)	330 (25.4)			
Other/ unknown (%)	22 (7.3)	16 (7.1)	89 (18.4)			21 (11.4)	7 (11.9)	151 (11.6)			
Disease severity											
Admitted (%)	39 (13)	22 (9.8)	83 (17.1)	.13	***.012***	26 (14.1)	4 (6.8)	139 (10.7)	.17	.51	.6
ICU level care (%)	13 (4.3)	9 (4)	28 (5.8)	.4	.37	8 (4.3)	2 (3.4)	46 (3.5)	.53	1	1
Death (%)	5 (1.7)	3 (1.3)	15 (3.1)	.25	.2	4 (2.2)	0	12 (0.9)	.13	1	1
Comorbidities											
Hypertension (%)	124 (41.3)	87 (38.9)	106 (21.9)	***.00001***	***<.00001***	91 (49.5)	26 (44.1)	369 (28.4)	***<.00001***	***.0125***	.55
Respiratory failure (%)	32 (10.7)	21 (9.4)	50 (10.3)	.90	.79	24 (13)	7 (11.9)	135 (10.4)	.307	.66	.6
Pregnancy (%)	18 (6)	14 (6.25)	35 (7.2)	.56	.75	13 (7.1)	7 (11.9)	105 (8.1)	.77	.33	.16
Lung disease (%)	50 (16.7)	33 (14.7)	74 (15.3)	.60	.91	47 (25.5)	14 (23.7)	311 (23.9)	.65	1	.12
Kidney disease (%)	43 (14.3)	29 (12.9)	39 (8)	***.006***	.05	45 (24.5)	13 (22)	159 (12.2)	***0***	***.042***	.1
Immunosuppression (%)	65 (21.7)	46 (20.5)	52 (10.7)	***.00***	***.001***	53 (28.8)	15 (25.4)	198 (15.3)	***0***	***.044***	.5
Diabetes (%)	61 (20.3)	40 (17.9)	47 (9.7)	***.00***	***.003***	51 (27.7)	13 (22)	184 (14.2)	***0***	.127	.5
Heart failure (%)	25 (8.3)	16 (7.1)	19 (3.9)	***.010***	.09	29 (15.8)	10 (16.9)	91 (7)	***.0002***	***.0096***	***.04***
Atrial fibrillation (%)	25 (8.3)	14 (6.25)	18 (3.7)	***.009***	.17	19 (10.3)	5 (8.5)	46 (3.5)	***.0002***	.07	.56
Smoker (%)	25 (8.3)	19 (8.5)	47 (9.7)	.60	.68	25 (13.6)	5 (8.5)	187 (14.4)	.8227	.25	1
Cerebrovascular disease (%)	31 (10.3)	18 (8)	22 (4.5)	***.002***	.08	26 (14.1)	5 (8.5)	78 (6)	***.0003***	.40	1
Cancer (%)	113 (37.7)	90 (40.2)	62 (12.8)	***<0.00001***	***<.00001***	87 (47.3)	31 (52.5)	231 (17.8)	***<.00001***	***<.00001***	.1
Coronary artery disease (%)	50 (16.7)	34 (15.2)	43 (8.9)	***.0014***	***.0140***	55 (29.9)	18 (30.5)	181 (13.9)	***<.00001***	***.002***	***.013***
								1 with unknown gender			

All positives after vaccination includes any patient who received vaccination prior to the positive test result. True breakthrough infections were based on the Centers for Disease Control and Prevention (CDC) definition to include positives more than 14 days after the second dose for Pfizer/BioNTech BNT162b2 or Moderna mRNA-1273 or 14 days after the J&J/Janssen shot. Statistics for ages and median days after vaccination were calculated by *t* test, and all other statistics were calculated by χ^2^ test.

Abbreviations: ICU, intensive care unit; SD, standard deviation.

### Delta and Alpha Variants Cycle Threshold (Ct) Values in Upper Respiratory Samples

**Figure 2.**
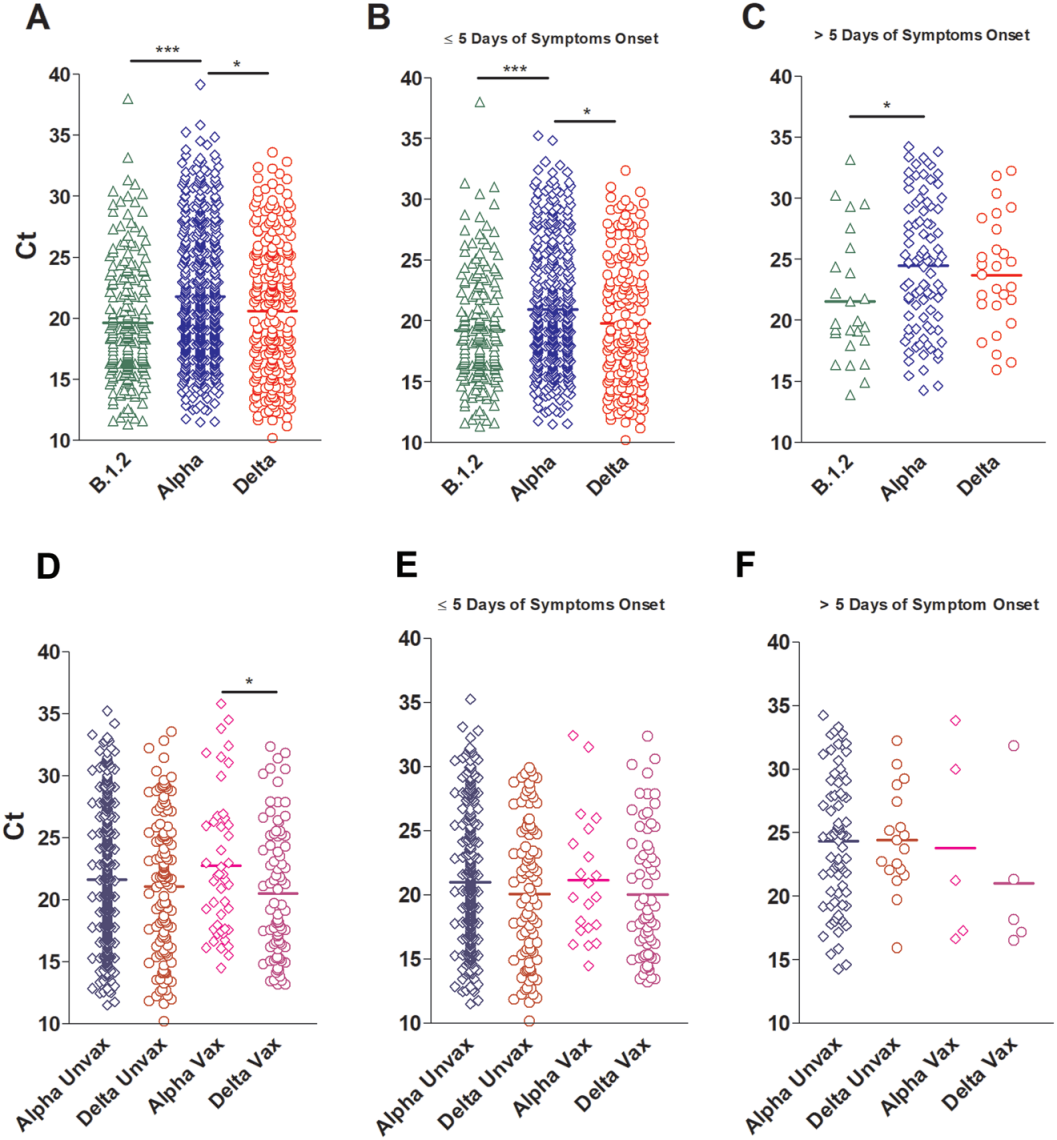
Delta and Alpha variants Ct values in upper respiratory samples. Abbreviations: Ct, cycle threshold; unvax, unvaccinated; vax, vaccinated.

To determine if the Ct values in respiratory specimens were different between Alpha, Delta, and B.1.2 variants, we compared the Ct values available for each group (N: Alpha = 564, Delta = 251, B.1.2 = 224) and associated the Ct values to the days after the onset of symptoms for symptomatic patients (N: Alpha = 511, Delta = 215, B.1.2 = 214). The mean Ct values for the Delta and B.1.2 variants were significantly lower when compared to the Alpha variant (20.61 vs 19.62 vs 21.77, respectively, Figure 2A). Similar trends were noted when Ct values were associated with samples collected within 5 days or less from symptoms onset (mean Ct for Alpha 20.98 vs 19.79 for Delta vs 19.25 for B.1.2, respectively, Figure 2B). For samples collected more than 5 days from symptoms, mean Ct values of the Alpha was significantly higher than the B.1.2 (24.5 vs 21.58, respectively, *P* < .05, Figure 2C). Supplementary Figure 1 confirms unbiased distribution of the data collected in correlation to days of the onset of symptoms for each group.

Mean Ct values were significantly lower in Delta versus Alpha variant vaccine breakthrough groups (20.51 vs 22.76, *P* < .05) (Figure 2D), but no significant differences were observed between Ct values from vaccinated and unvaccinated individuals infected by either variant (Figure 2E and 2F).

### Recovery of Infectious Virus in Delta versus Alpha groups

**Figure 3.**
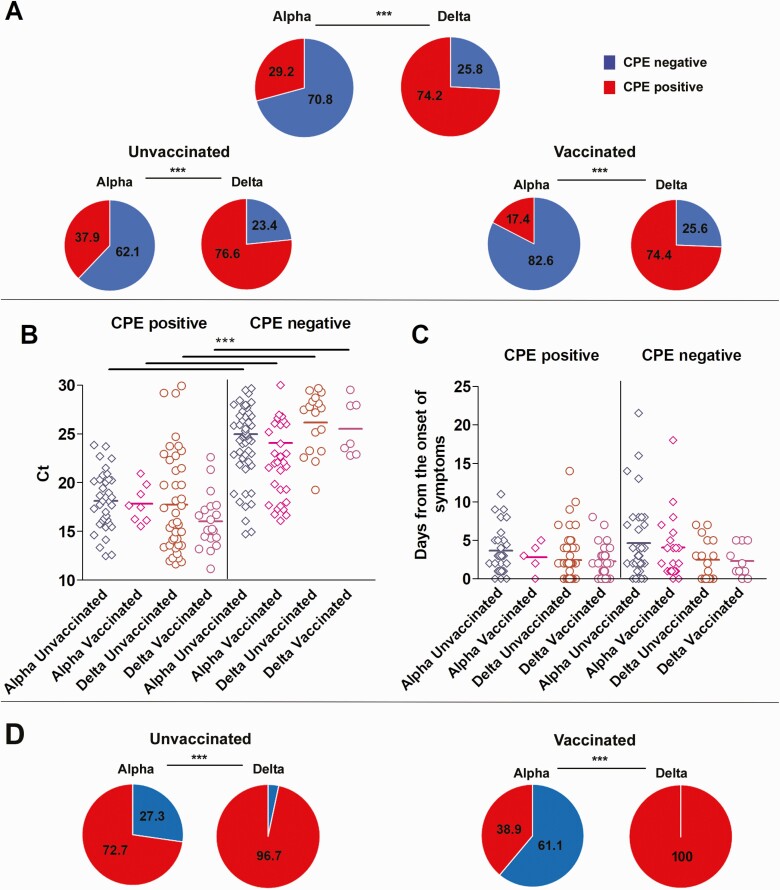
Recovery of infectious virus in Delta versus Alpha groups. Abbreviation: CPE, cytopathic effect; Ct, cycle threshold.

Samples from a total of 154 Alpha (95 from unvaccinated, 13 from partially or unknown vaccination status, and 46 from vaccine breakthrough infections) and 128 Delta (77 unvaccinated, 12 from partially or unknown vaccination status, and 39 from vaccine breakthrough infections) were used to inoculate Vero-TMPRSS2 cells. Significantly more specimens with Delta variants had infectious virus present compared to specimens containing Alpha variants (Delta 74.2%, Alpha 29.2%; Figure 3A, *P* < .00001). Specimens from the fully vaccinated Alpha group showed significant reduction in the recovery of infectious virus as compared to the unvaccinated Alpha group (17.4% vs 37.9%, *P* = .02, Figure 3A), but in the Delta groups there were nearly equivalent specimens with infectious virus (76.6% vs 74.4%, Figure 3A). A significant increase in the recovery of infectious virus from specimens with the Delta variant as compared to the Alpha variant was noted for both unvaccinated (76.6% vs 37.9%, *P* < .00001) and fully vaccinated (74.4% vs 17.4%, *P* < .00001) groups (Figure 3A). The mean Ct value for specimens associated with infectious virus in all groups was significantly lower than groups without infectious virus (*P* < .0001) but no differences in mean Ct values were noted between Alpha and Delta vaccinated and unvaccinated groups in infectious virus positive or negative groups (Figure 3B).No significant differences were noted in the specimen collection time frame in relation to the onset of symptoms in all groups (Figure 3C). Because lower Ct values have been associated with positive recovery of infectious virus on cell culture, we compared samples with Ct values <20 for both Delta and Alpha vaccinated and unvaccinated groups to control for this confounding factor (Alpha N = 51, Delta N = 47). Delta infection was associated with a significant increase in samples with positive infectious virus compared to Alpha for both fully vaccinated (100% vs 38.9%, *P* < .00001) and unvaccinated (96.7% vs 72.7%, *P* < .00001) cohorts (Figure 3D).

### Localized SARS-CoV-2 IgG in the Delta versus the Alpha Groups

**Figure 4.**
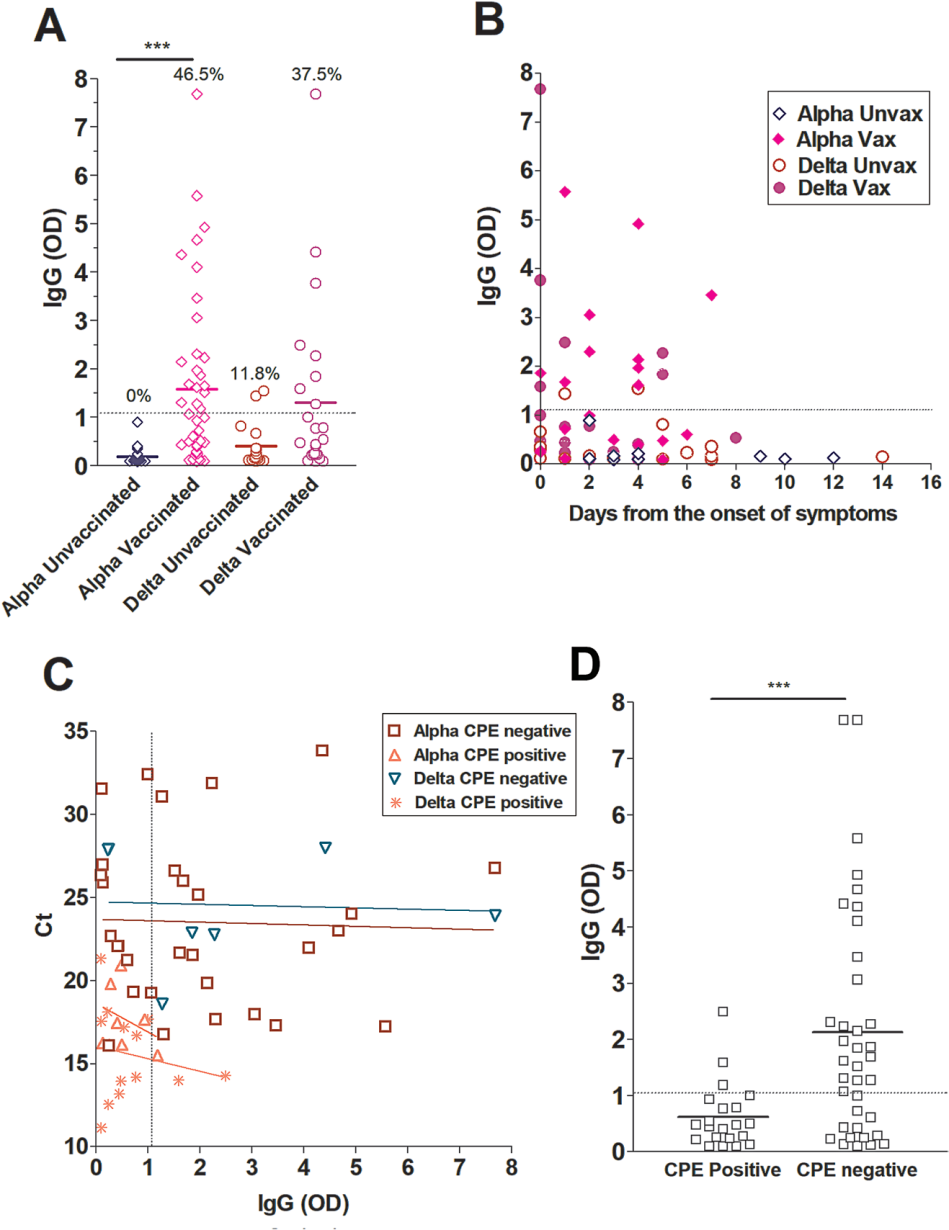
Localized SARS-CoV-2 IgG in the Delta versus the Alpha groups. Abbreviations: CPE, cytopathic effect; Ct, cycle threshold; IgG, immunoglobulin G; OD, optical density; SARS-CoV-2, severe acute respiratory syndrome coronavirus 2; unvax, unvaccinated; vax, vaccinated.

To assess the relationship between upper respiratory tract SARS-CoV-2 IgG levels in fully vaccinated patients and the recovery of infectious virus, ELISA was performed on upper respiratory samples from fully vaccinated individuals infected with Alpha (N = 43) or Delta (N = 24) variants as well as control unvaccinated but infected groups (Alpha, N = 30 and Delta, N = 17). A significant increase in localized IgG levels was observed in vaccinated versus unvaccinated individuals infected with the Alpha variant (Alpha unvaccinated, 0% positives, vaccinated 46.5% positives, *P* < .0001). More vaccinated individuals infected by the Delta variant showed detectable upper respiratory tract IgG but the mean IgG levels were not different between the groups (Delta unvaccinated 11.8% positives, vaccinated 37.5% positives) (Figure 4A). The distribution of sample collection in relation to the time from the onset of symptoms was similar between the groups (Figure 4B). Specimens from vaccine breakthrough patients from both Alpha and Delta variants demonstrated an inverse correlation between upper respiratory tract IgG levels and the recovery of infectious virus on cell culture, regardless of Ct value (Figure 4C and 4D).

## DISCUSSION

In this study, we showed that breakthrough infections with the Delta variant were associated with higher rates of recovery of infectious virus from respiratory samples compared to the Alpha. This was associated with a significant increase in the median time after receiving the vaccine, which was likely associated with the temporal circulation of each with the rollout of vaccination. The recovery of infectious virus correlated with lower localized IgG levels in the respiratory tracts. The disease in fully vaccinated individuals was not more severe in the Delta versus the Alpha infected groups and in general, breakthrough infections associated with significant increases in certain comorbidities. Taken together, our study indicates that the reduction in antibodies associated with time since vaccination might increase the likelihood of symptomatic disease and infectiousness but not severe disease.

The main observation in this cohort was the association of the Delta with more breakthrough infections and the significant increase in the days since receiving the vaccine in these cases. Breakthrough infections with the Delta variant were also associated with a slight increase in viral loads and marked increase in the recovery of infectious virus from infected vaccinated and unvaccinated patients when compared to the Alpha variant. We previously showed that vaccination was associated with reduction of the recovery of infectious virus on cell culture in a cohort primarily infected with the Alpha variant between January 2021 and May 2021, and this was associated with higher upper respiratory tract IgG levels [24]. The increase in vaccine breakthrough infections seen with Delta could be due to waning immune responses as a result of the extended time post vaccination. Interestingly, the recovery of infectious virus was notable in samples with negative or low upper respiratory tract IgG levels and was more prominent with the Delta group, a correlation that was independent of the relative viral loads in the specimens. Our data are consistent with a recent observation from Vietnam that associated Delta breakthrough infections with lower levels of neutralizing antibodies induced by vaccination and a study from Wisconsin that showed high levels of infectious virus recovery from vaccinated patients [16, 17]. Our data also suggest that increasing the upper respiratory tract IgG—perhaps through booster vaccinations—could help reduce transmission and symptomatic infections.

The higher recovery of infectious virus from the Delta group regardless of vaccination status, which did not correlate to differences in the relative Ct values of the samples, suggests increased fitness of Delta. Changes within the spike protein of the Delta variant are thought to lead to enhanced binding to the host cell receptor (ACE2) and the S: P681R change in particular might increase the S protein cleavage efficiency allowing for more efficient entry [25]. In addition, the S: L452R could contribute to the noticeable reduction in neutralization by serum antibodies and monoclonal antibodies [26–28]. However, mutations in other parts of the viral genome may contribute to virus fitness and immune evasion as previously described for SARS-CoV-2 [29]. Our use of Vero-TMPRSS2 cells to perform virus isolation may also be important in detecting individuals with infectious Delta variant in their nasal swab specimen.

The limitations of our study include the infrequent specimens collected after 5 days of symptoms onset for the vaccine breakthrough groups, the retrospective nature of data and sample collection, and the infrequent missing data from patients’ electronic medical charts. In addition, the phenotypes with cell culture experiments are usually dependent on the cell lines used, even though Vero-TMPRSS-2 cells have been shown to enhance the isolation of SARS-CoV-2 [30]. Moreover, the lack of serum and localized SARS-CoV-2 IgG data prior to infection for vaccine breakthrough cases in our cohort does not allow for the differentiation between waning immune responses and low initial responses to vaccines. In addition, quantification of viruses and quantitative antibody neutralization assays from clinical samples were not conducted as a part of this study. It is important to note that measures of infection control, including masking and social distancing, were different from the summer of 2021 when Delta predominated, compared to the winter of 2021 when Alpha dominated, a variable that our study cannot control for.

We hypothesize that the increase in time since receiving the vaccines combined with increased fitness of the Delta variant predisposes both vaccinated and unvaccinated individuals to symptomatic SARS-CoV-2 infections that are associated with higher viral loads and transmission. Yet vaccinated patients infected with the Delta had comparable admissions and mortality when compared to the Alpha infected vaccinated patients, suggesting continued protection from severe disease.

## Supplementary Data

Supplementary materials are available at *Clinical Infectious Diseases* online. Consisting of data provided by the authors to benefit the reader, the posted materials are not copyedited and are the sole responsibility of the authors, so questions or comments should be addressed to the corresponding author.

ciab986_suppl_Supplementary_Figure-S1

## Notes

***Acknowledgements.*** This study was only possible with the unique efforts of the Johns Hopkins Clinical Microbiology Laboratory faculty and staff. H. H. M. is supported by the HIV Prevention Trials Network (HPTN) sponsored by the National Institute of Allergy and Infectious Diseases (NIAID). Funding was provided by the Johns Hopkins Center of Excellence in Influenza Research and Surveillance (grant number HHSN272201400007C), National Institute on Drug Abuse, National Institute of Mental Health, and Office of AIDS Research, of the National Institute of Health (NIH), Department of Health and Human Services (DHHS) (grant number UM1 AI068613), the NIH RADx-Tech program (grant number 3U54HL143541-02S2), NIHRADx-UP initiative (grant number R01 DA045556-04S1), Centers for Disease Control and Prevention (CDC) (contract 75D30121C11061), the Johns Hopkins University President’s Fund Research Response, the Johns Hopkins department of Pathology, and the Maryland Department of Health. E. K. was supported by CDC MInD-Healthcare Program (grant number U01CK000589). The views expressed in this manuscript are those of the authors and do not necessarily represent the views of the National Institute of Biomedical Imaging and Bioengineering; the National Heart, Lung, and Blood Institute; the NIH, or the US DHHS. Whole genome data were made available publicly and raw genomic data requests could be directed to H. H. M.

***Financial support.*** NIH/NIAID Center of Excellence in Influenza Research and Surveillance contract HHS N2772201400007C (provided funding to support the virus isolation efforts), Johns Hopkins University, Maryland Department of Health, CDC contract 75D30121C11061. H. H. M. reports the following support: CDC BAA (grant number 75D301-21-C-11061), Maryland Department of Health, and The Johns Hopkins University.

***Potential conflicts of interest.*** E. Y. K. reports support from Center for Disease Control and Prevention. H. H. M. reports the following support: DiaSorin Molecular, NIH/ CDC/ BARDA, FCDP Grant, Johns Hopkins University, BioRad (grant numbers NIH 4UH3CA211396-03, NIH R01 CA243393, and NIH R01 CA243393); reports receipt of research contract from the following BioRad, DiaSorin, and Hologic.

All other authors report no potential conflicts. All authors have submitted the ICMJE Form for Disclosure of Potential Conflicts of Interest. Conflicts that the editors consider relevant to the content of the manuscript have been disclosed.

## References

[1] CDC. SARS-CoV-2 Variant Classifications and Definitions. (2021). Centers for Disease Control and Prevention. Available at: https://www.cdc.gov/coronavirus/2019-ncov/cases-updates/variant-surveillance/variant-info.html#Concern. Accessed 12 October 2021.

[2] Public-Health-England. Investigation of novel SARS-CoV-2 variants of concern. 2021. Available at: https://www.gov.uk/government/publications/investigation-of-novel-sars-cov-2-variant-variant-of-concern-20201201. Accessed 12 October 2021.

[3] Áine O’Toole VH , Oliver G. Pybus AW, et al; The COVID-19 Genomics UK (COG-UK) consortium, Network for Genomic Surveillance in South Africa (NGS-SA), Brazil-UK CADDE Genomic Network, Swiss Viollier Sequencing Consortium, SEARCH Alliance San Diego, National Virus Reference Laboratory, SeqCOVID-Spain, Danish Covid-19 Genome Consortium (DCGC), Communicable Diseases Genomic Network (CDGN), Dutch National SARS-CoV-2 surveillance program,#, Division of Emerging Infectious Diseases KDCA. Tracking the international spread of SARS-CoV-2 lineages B.1.1.7 and B.1.351/501Y-V2. 2021. Available at: https://virological.org/t/tracking-the-international-spread-of-sars-cov-2-lineages-b-1-1-7-and-b-1-351-501y-v2/592. Accessed 28 September 2021.

[4] Morris CP , Luo CH, Amadi A, et al An update on SARS-CoV-2 diversity in the United States national capital region: evolution of novel and variants of concern. Clin Infect Dis 2021. doi:10.1093/cid/ciab636. PMC8406876 34272947

[5] Davies NG , Abbott S, Barnard RC, et al Estimated transmissibility and impact of SARS-CoV-2 lineage B.1.1.7 in England. Science 2021; 372:eabg3055. 33658326 10.1126/science.abg3055 PMC8128288

[6] Davies NG , Jarvis CI, van Zandvoort K, et al Increased mortality in community-tested cases of SARS-CoV-2 lineage B.1.1.7. Nature 2021. doi:10.1038/s41586-021-03426-1 PMC9170116 33723411

[7] Frampton D , Rampling T, Cross A, et al Genomic characteristics and clinical effect of the emergent SARS-CoV-2 B.1.1.7 lineage in London, UK: a whole-genome sequencing and hospital-based cohort study. Lancet Infect Dis 2021. doi:10.1016/S1473-3099(21)00170-5. PMC8041359 33857406

[8] Chia PY , Xiang Ong SW, Chiew CJ, et al Virological and serological kinetics of SARS-CoV-2 Delta variant vaccine-breakthrough infections: a multi-center cohort study. Clin Microbiol Infect 2021. doi:10.1016/j.cmi.2021.11.010 PMC8608661 34826623

[9] Brown CM , Vostok J, Johnson H, et al Outbreak of SARS-CoV-2 infections, including COVID-19 vaccine breakthrough infections, associated with large public gatherings - Barnstable County, Massachusetts, July 2021. MMWR Morb Mortal Wkly Rep 2021; 70:1059–62. 34351882 10.15585/mmwr.mm7031e2 PMC8367314

[10] Christensen PA , Olsen RJ, Long SW, et al Delta Variants of SARS-CoV-2 Cause Significantly Increased Vaccine Breakthrough COVID-19 Cases in Houston, Texas. Am J Clin Pathol 2021. doi:10.1016/j.ajpath.2021.10.019. PMC8580569 34774517

[11] Sheikh A , McMenamin J, Taylor B, Robertson C, Public Health S, the EIIC. SARS-CoV-2 Delta VOC in Scotland: demographics, risk of hospital admission, and vaccine effectiveness. Lancet 2021; 397:2461–2. 34139198 10.1016/S0140-6736(21)01358-1 PMC8201647

[12] Lopez Bernal J , Andrews N, Gower C, et al Effectiveness of Covid-19 vaccines against the B.1.617.2 (Delta) variant. N Engl J Med 2021. doi:10.1056/NEJMoa2108891. PMC8314739 34289274

[13] Thompson MG , Burgess JL, Naleway AL, et al Prevention and attenuation of Covid-19 with the BNT162b2 and mRNA-1273 vaccines. N Engl J Med 2021; 385:320–9. 34192428 10.1056/NEJMoa2107058 PMC8262622

[14] Teyssou E , Soulie C, Visseaux B, et al The 501Y.V2 SARS-CoV-2 variant has an intermediate viral load between the 501Y.V1 and the historical variants in nasopharyngeal samples from newly diagnosed COVID-19 patients. J Infect 2021; 83:119–45. doi:10.1101/2021.03.21.21253498:2021.03.21.21253498. PMC8080495 33932451

[15] Petra M , Steven K, Mahesh Shanker D, et al; The Indian S-C-GC, Citiid-Nihr BioResource Covid-19 Collaboration AM. Nature Portfolio. 2021. doi:10.21203/rs.3.rs-637724/v1.

[16] Riemersma KK , Grogan BE, Kita-Yarbro A, et al Shedding of infectious SARS-CoV-2 despite vaccination when the delta variant is prevalent - Wisconsin, July 2021. medRxiv 2021. doi:10.1101/2021.07.31.21261387

[17] Chau NVV , Ngoc NM, Nguyet LA, et al An observational study of breakthrough SARS-CoV-2 Delta variant infections among vaccinated healthcare workers in Vietnam. EClinicalMedicine 2021; 41:101143. https://papers.ssrn.com/sol3/papers.cfm?abstract_id=3897733. 34608454 10.1016/j.eclinm.2021.101143 PMC8481205

[18] Jarrett J , Uhteg K, Forman MS, et al Clinical performance of the GenMark Dx ePlex respiratory pathogen panels for upper and lower respiratory tract infections. J Clin Virol 2021;135:104737. 33497932 10.1016/j.jcv.2021.104737

[19] Mostafa HH , Carroll KC, Hicken R, et al Multi-center evaluation of the cepheid Xpert(R) Xpress SARS-CoV-2/Flu/RSV test. J Clin Microbiol 2020. doi:10.1128/JCM.02955-20 PMC8106732 33298613

[20] Mostafa HH , Hardick J, Morehead E, Miller JA, Gaydos CA, Manabe YC. Comparison of the analytical sensitivity of seven commonly used commercial SARS-CoV-2 automated molecular assays. J Clin Virol 2020; 130:78. 10.1016/j.jcv.2020.104578 PMC7405824 32777761

[21] Uhteg K , Jarrett J, Richards M, et al Comparing the analytical performance of three SARS-CoV-2 molecular diagnostic assays. J Clin Virol 2020; 127:104384. 32361285 10.1016/j.jcv.2020.104384 PMC7194987

[22] Thielen PM , Wohl S, Mehoke T, et al Genomic diversity of SARS-CoV-2 during early introduction into the Baltimore-Washington metropolitan area. JCI Insight 2021; 6:e144350. doi:10.1172/jci.insight.144350. PMC8026189 33749660

[23] Gniazdowski V , Morris CP, Wohl S, et al Repeat COVID-19 molecular testing: correlation of SARS-CoV-2 culture with molecular assays and cycle thresholds. Clin Infect Dis 2020. doi:10.1093/cid/ciaa1616. PMC7665437 33104776

[24] Mostafa HH , Luo CH, Morris CP, et al SARS-CoV-2 infections in mRNA vaccinated individuals are biased for viruses encoding spike E484K and associated with reduced infectious virus loads that correlate with respiratory antiviral IgG levels. medRxiv 2021. doi:10.1101/2021.07.05.21259105:2021.07.05.21259105. PMC8979609 35398602

[25] Scudellari M. How the coronavirus infects cells - and why Delta is so dangerous. Nature 2021; 595:640–4. 34321669 10.1038/d41586-021-02039-y

[26] Lucas C , Vogels CBF, Yildirim I, et al Impact of circulating SARS-CoV-2 variants on mRNA vaccine-induced immunity in uninfected and previously infected individuals. medRxiv 2021. doi:10.1101/2021.07.14.21260307:2021.07.14.21260307. PMC9348899 34634791

[27] Planas D , Veyer D, Baidaliuk A, et al Reduced sensitivity of SARS-CoV-2 variant Delta to antibody neutralization. Nature 2021; 596:276–80. 34237773 10.1038/s41586-021-03777-9

[28] Lazarevic I , Pravica V, Miljanovic D, Cupic M. Immune evasion of SARS-CoV-2 emerging variants: what have we learnt so far? Viruses 2021; 13:1192. doi:10.3390/v13071192 34206453 PMC8310325

[29] Graham RL , Sparks JS, Eckerle LD, Sims AC, Denison MR. SARS coronavirus replicase proteins in pathogenesis. Virus Res 2008; 133:88–100. 17397959 10.1016/j.virusres.2007.02.017 PMC2637536

[30] Matsuyama S , Nao N, Shirato K, et al Enhanced isolation of SARS-CoV-2 by TMPRSS2-expressing cells. Proc Natl Acad Sci U S A 2020; 117:7001–3. doi:10.1073/pnas.2002589117 32165541 PMC7132130

