# Supplementary figures and images for: Infection With the Severe Acute Respiratory Syndrome Coronavirus 2 (SARS-CoV-2) Delta Variant Is Associated With Higher Recovery of Infectious Virus Compared to the Alpha Variant in Both Unvaccinated and Vaccinated Individuals
Source: Clin Infect Dis. 2021 Dec 18;75(1):e715–25. doi: 10.1093/cid/ciab986 (PMC8903351; doi:10.1093/cid/ciab986)

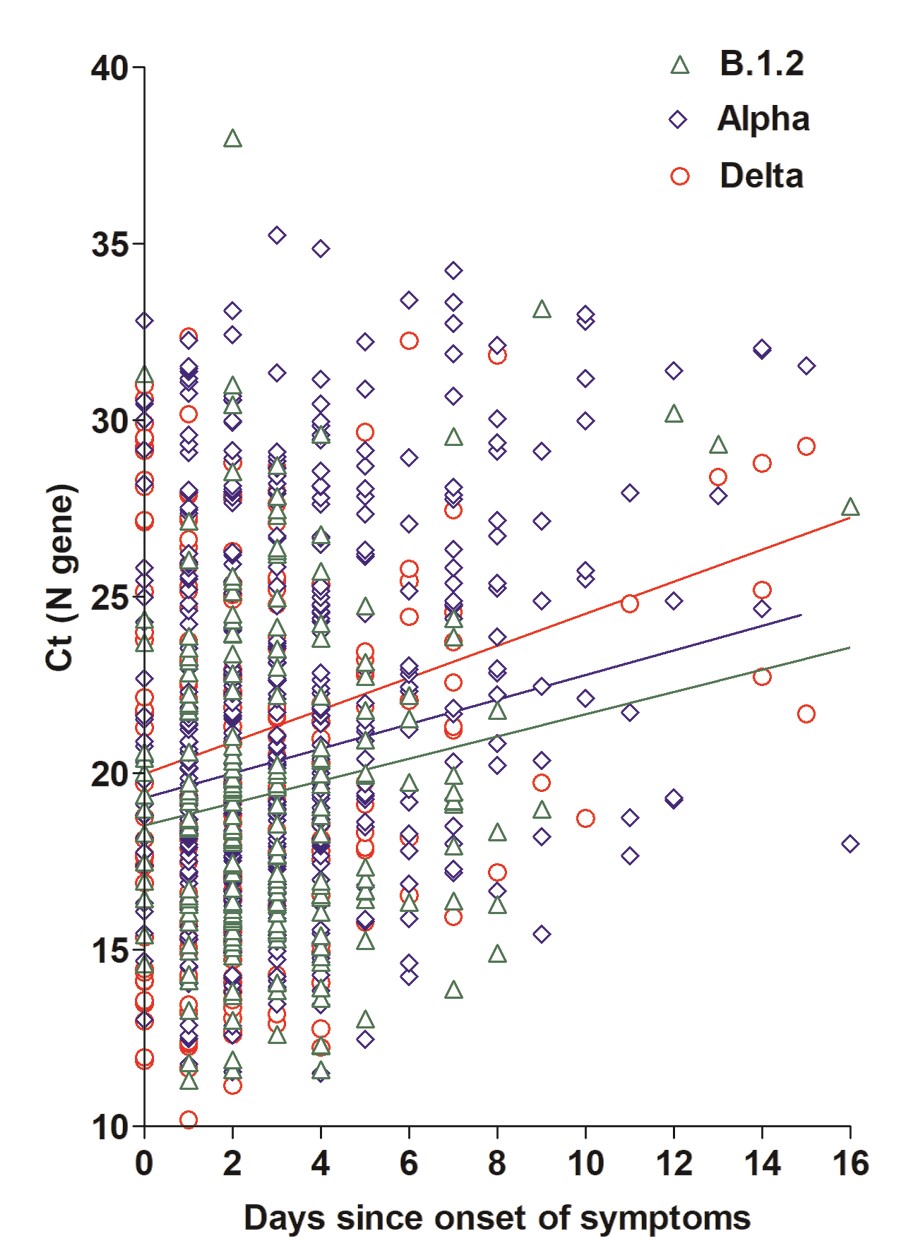

Supplement: ciab986_suppl_Supplementary_Figure-S1 [file ciab986_suppl_supplementary_figure-s1.jpeg]
